# Polymerization of novel methacrylated anthraquinone dyes

**DOI:** 10.3762/bjoc.9.48

**Published:** 2013-02-28

**Authors:** Christian Dollendorf, Susanne Katharina Kreth, Soo Whan Choi, Helmut Ritter

**Affiliations:** 1 Institute for Organic and Macromolecular Chemistry II, Heinrich-Heine-Universität Düsseldorf, Universitätsstraße 1, 40225 Düsseldorf, Germany

**Keywords:** anthraquinone, monomeric dyes, red, green, blue dyes, polymer

## Abstract

A new series of polymerizable methacrylated anthraquinone dyes has been synthesized by nucleophilic aromatic substitution reactions and subsequent methacrylation. Thereby, green 5,8-bis(4-(2-methacryloxyethyl)phenylamino)-1,4-dihydroxyanthraquinone (**2**), blue 1,4-bis(4-((2-methacryloxyethyl)oxy)phenylamino)anthraquinone (**6**) and red 1-((2-methacryloxy-1,1-dimethylethyl)amino)anthraquinone (**12**), as well as 1-((1,3-dimethacryloxy-2-methylpropan-2-yl)amino)anthraquinone (**15**) were obtained. By mixing of these brilliant dyes in different ratios and concentrations, a broad color spectrum can be generated. After methacrylation, the monomeric dyes can be covalently emplaced into several copolymers. Due to two polymerizable functionalities, they can act as cross-linking agents. Thus, diffusion out of the polymer can be avoided, which increases the physiological compatibility and makes the dyes promising compounds for medical applications, such as iris implants.

## Introduction

Anthraquinone and its derivatives bearing hydroxy and amino moieties are of remarkable importance in pharmacological, biochemical and dye industries [1–2]. They have been described as important compounds for decades [3–6] and are used for the coloration of cotton and cellulose fibers as well as for synthetic materials such as polyamides [7]. Besides azo dyes, they represent the second largest class of textile pigments [8], and unlike these dyes, anthraquinone derivatives are resistant against degradation due to their aromatic structure [9]. As the syntheses of these dyes are more complicated, their production costs are higher in comparison to azo dyes. Therefore, anthraquinone dyes are only used if the required properties and colors are remarkable or when the desired colors cannot be obtained easily by the use of azo dyes, especially in the case of bright blue shades. Anthraquinone dyes can be obtained synthetically and by isolation from fungi such as *Dermocybe sanguinea* or other fungal species [5,10–13], to give them a higher compatibility with the environment and an improved availability [14–16].

Coloration of polymers is usually achieved by mixing dyes or pigments with (other) polymeric ingredients. In many cases, the colors rapidly fade or change as well as lose their mechanical properties when exposed to sunlight. More stable polymers can be prepared by the reaction of polymerizable dyes with appropriate comonomers. In the past, these materials have been widely used because of their photo- and thermostability, their strong fluorescence, or their resistance to water and other solvents [7,17–18].

The purpose of this work was to create dyes of brilliant color modified with polymerizable groups, to enable the preparation of polymeric materials with covalently emplaced dyes. A broad range of the color spectrum can be produced by mixing three colors such as blue, green and red, which were synthesized in this work [19–24]. For example, colored polymers can be used for medical applications such as iris implants, where damaged irides are replenished with polymeric replications [25–27].

## Results and Discussion

**Scheme 1:**
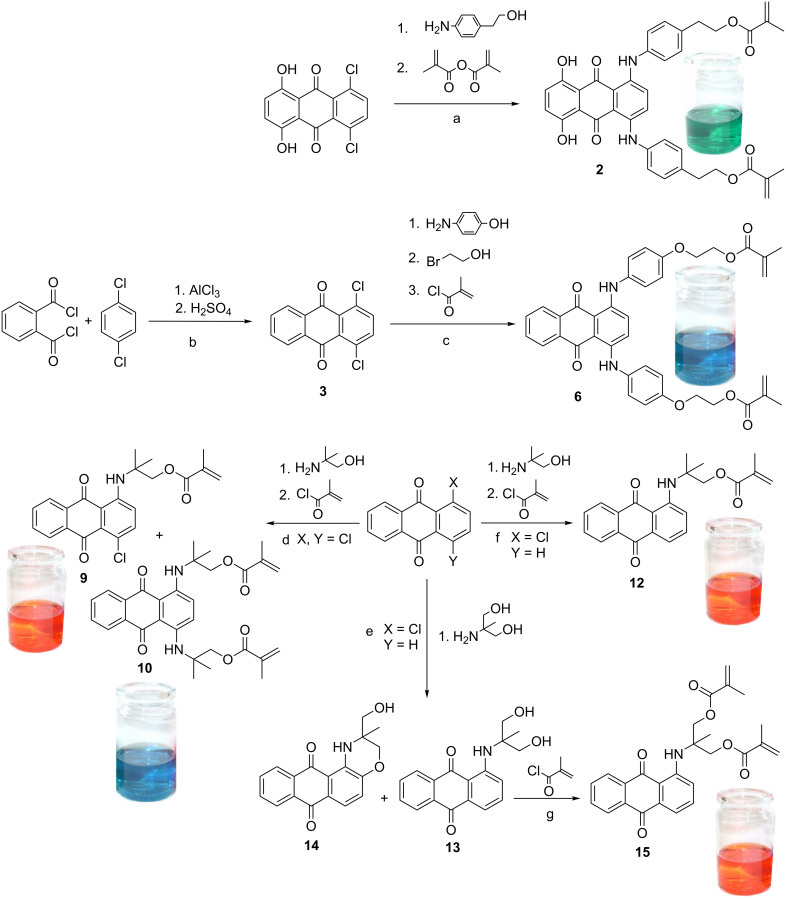
Synthesis of red (**9**, **12**, **15**), blue (**6**, **10**) and green (**2**) polymerizable dyes.

Starting from commercially available anthraquinone-derivatives, various polymerizable dyes were synthesized. Three colors (green, blue, red) were obtained (Scheme 1).

### Polymerizable green dye **2** based on anthraquinone

The synthesis of the green anthraquinone based monomeric dye **2** is shown in Scheme 1, step a. 5,8-Dichloro-1,4-dihydroxyanthraquinone was first subjected to a nucleophilic substitution reaction with 2-(4-aminophenyl)ethanol to give a green-colored double-substituted intermediate 5,8-bis(4-(2-hydroxyethyl)phenylamino)-1,4-dihydroxyanthraquinone (**1**) in 50% overall yield. According to the literature, reactions can be accelerated with improved yields by microwave assistance [28–29]. After microwave-assisted methacrylation with methacrylic anhydride the polymerizable anthraquinone based monomeric dye **2** was obtained. Due to steric reasons, methacrylic anhydride is less reactive than the corresponding chloride and therefore selective for the methacrylation of the aliphatic hydroxy groups [25]. In this case, the use of methacryloyl chloride always leads to the formation of several side-products. As cross-linking agent, **2** can be copolymerized with other methacrylate- or acrylate-comonomers, to build up macromolecules bearing covalently emplaced dye derivatives.

**Figure 1:**
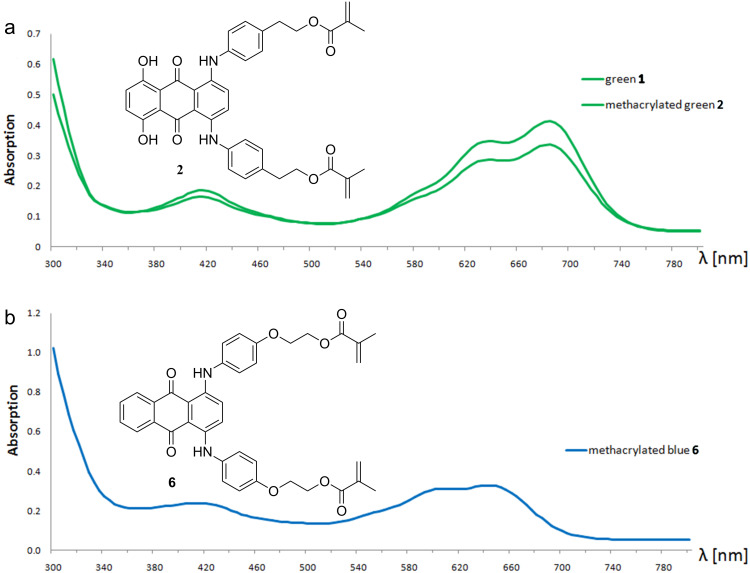
Visible spectra of the polymerizable dyes green **1**/**2** (a) and blue **6** (b).

The UV–vis spectra of **1** and **2** (Figure 1a) in each case show three absorption maxima (416, 636 and 684 nm), which can be found in the violet and red color ranges. A combination of these two colors appears purple, the complementary color to green. Methacrylation of **1** does not change the chromophore and therefore the absorption maxima. The change in the absorption behavior of **2** compared to 5,8-dichloro-1,4-dihydroxyanthraquinone (λ_max_ = 500 nm) is a result of the enlargement of the π-electron system as aromatic moieties are introduced. This leads to absorption at longer wavelengths (bathochromic shift). Thus, the challenge of creating a green dye is to realize selective absorption in two different areas of the visible spectrum.

### Polymerizable blue dye **6** based on anthraquinone

For the synthesis of the blue anthraquinone based monomeric dye **6**, the precursor 1,4-dichloroanthraquinone (**3**) was prepared by the reaction of 1,4-dichlorobenzene with phthalic dichloride by Friedel–Crafts acylation followed by an acidic ring closure (Scheme 1, step b). In further reaction steps, 1,4-dichloroanthraquinone (**3**) and hydroxyaniline were reacted to obtain 1,4-bis(4-hydroxyphenylamino)anthraquinone (**4**). Subsequent reaction with 2-bromoethanol gave 1,4-bis(4-((2-hydroxyethyl)oxy)phenylamino)anthraquinone (**5**). In order to introduce polymerizable functionalities, **5** was treated with methacrylic chloride to give 1,4-bis(4-((2-methacryloxyethyl)oxy)phenylamino)anthraquinone (**6**) (Scheme 1, step c).

The obtained blue chromophore **6** shows absorption maxima at 408, 600 and 644 nm in the UV–vis spectrum (Figure 1b). These maxima are located in the purple, orange and red color ranges, which results in combination to give the color blue. Analogous to the green dye, methacrylation of **5** does not influence the absorption maxima.

### Polymerizable red dyes **9**, **12** and **15** based on anthraquinone

A red monomeric dye **9** with non-aromatic amines as functional moieties, which showed similar properties, was also synthesized starting from anthraquinone. 1,1-Dimethyl-2-hydroxyethylamine exhibits two methyl groups in the α*-*position to the nitrogen atom, which shields the chromophoric amine. The absence of hydrogen atoms in the α*-*position also protects the amino group from further reactions. In the cases of the green and blue monomeric dyes this effect was achieved by introducing a benzene ring in the α*-*position [25].

**Figure 2:**
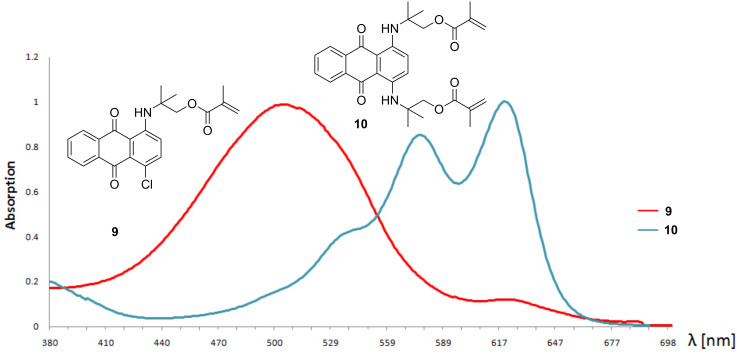
Visible spectra of red **9** and blue **10**.

1,4-Dichloroanthraquinone was treated with 1,1-dimethyl-2-hydroxyethylamine to form two products, namely 1-chloro-4-((2-hydroxy-1,1-dimethylethyl)amino)anthraquinone (**7**) and 1,4-bis((2-hydroxy-1,1-dimethylethyl)amino)anthraquinone (**8**). Both products can be separated by column chromatography and esterified with methacryloyl chloride to obtain polymerizable monomeric dyes **9** and **10** (Scheme 1, step d). The monosubstituted product **9** shows a characteristic red color and an absorption maximum at 506 nm, whereas the disubstituted product **10** is colored blue with absorption maxima at 574 and 618 nm (Figure 2).

The synthesis of a red polymerizable dye **12** without any side-products can be accomplished by the use of 1-chloroanthraquinone and 1,1-dimethyl-2-hydroxyethylamine giving 1-((1-hydroxy-2-methylpropane-2-yl)amino)anthraquinone (**11**) in good yields (60%). Subsequent esterification with methacryloyl chloride gives **12** [26–27]. This dye shows a characteristic red color with an absorption maximum at 506 nm, which is located in the green color range, the complementary color to red.

By introduction of a second polymerizable functionality, diffusion of incompletely polymerized dyes out of the polymer network can be avoided, which increases the physiological compatibility. This monomer can also be used as a cross-linking agent. For this purpose, 1-chloroanthraquinone was coupled with 2-amino-2-methyl-1,3-propanediol under argon atmosphere and esterified with methacryloyl chloride to obtain **15** (Scheme 1, step g), which absorbs light at 495 nm.

In the absence of argon, the formation of a side-product can be observed. An oxidative ring closure takes place during the reaction of 1-chloroanthraquinone and 2-amino-2-methyl-1,3-propanediol, which gives **14** as a side-product in low yield (6%). The yield can be increased by microwave assistance and the use of higher temperatures [27]. It can be assumed that the driving force for the ring closure is the formation of a six-membered ring; however, the mechanism has not been investigated in detail so far. Since the ring closure does not affect the spectral properties, **14** can still be used as a polymerizable red dye.

In this work, side-products derived from ring-closure reactions were only found when aliphatic amines with two carbon units between the amine and hydroxy functionality were used. In the synthesis of green and blue dyes, aromatic amines that cannot build six-membered rings were utilized. However, the ring-closure can be suppressed by using inert gas or adequate compounds such as 5-aminopentane-1-ol, for example [27].

### Polymerization and photosensitivity of the synthesized monomeric dyes **6**, **9** and **10**

**Figure 3:**
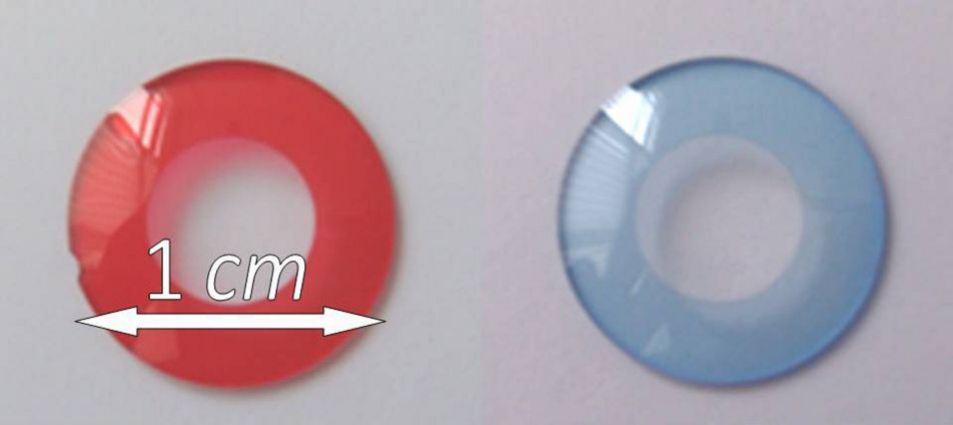
Sharpened blank of polymerized red **9** and blue **10** with HEMA, THFMA and EGDMA (left, right).

By way of example for copolymerization of two monomeric dyes, **9** or **10** were copolymerized with 2-hydroxyethylmethacrylate (2-HEMA), tetrahydrofurfuryl methacrylate (THFMA) and ethylene glycol dimethacrylate (EGDMA) as cross-linking agents in order to obtain materials with duroplastic properties and high resistance against aggressive biological media [28,30–32]. When the polymers were placed in water, even after several weeks, no diffusion of monomeric dyes out of the network was observed, as examined by UV–vis spectroscopy. This proves the covalent emplacement of the monomeric dyes in the polymer network. Figure 3 shows polymers containing **9** and **10** after sharpening for further application as iris implants.

**Figure 4:**
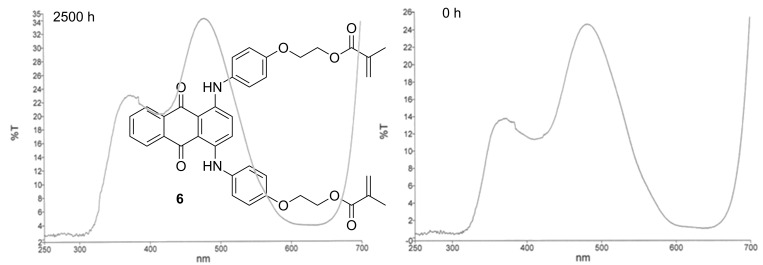
Sun-test results of **6**.

For use in medical applications like iris implants, the dyes and the corresponding polymers have to be biocompatible (ISO 11979-5) [33–35]. To analyze the photosensitivity of the materials, the dye-containing polymers were preserved in a sodium chloride solution at 35 °C and exposed to irradiation with visible light (3 mW/cm^2^ 2500 h) for 100 days. As an example, Figure 4 shows the transmission spectrum for the polymer containing cross-linker **6**. After 100 days, no changes in the transmission spectra could be observed, which proves the monomer dye stability and is also a hint toward its biocompatibility.

### Mixture of polymerizable dyes **2**, **6** and **15**

By mixing the synthesized blue, red and green dyes (**6**, **15** and **2**) in different ratios, individual color shades can be realized. This so-called RGB-color space can also be found in other color devices, such as monitors, scanners and color printers. A broad visible spectrum can be covered by mixing these three colors in different ratios [19–21].

The subjective color impression depends deeply on the used monomer concentration [36]. For example, the red dye **15** appears orange in high dilution, and the green dye **2** appears greenish-blue. At the same concentration, the blue dye **6** shows the lowest transparency. By mixing these colors, most of the blue-containing nuances only slightly differ. Due to the absorption of the broadest area in the visible spectrum and the lowest brilliance and transparency, the blue color masks the green and red one.

**Figure 5:**
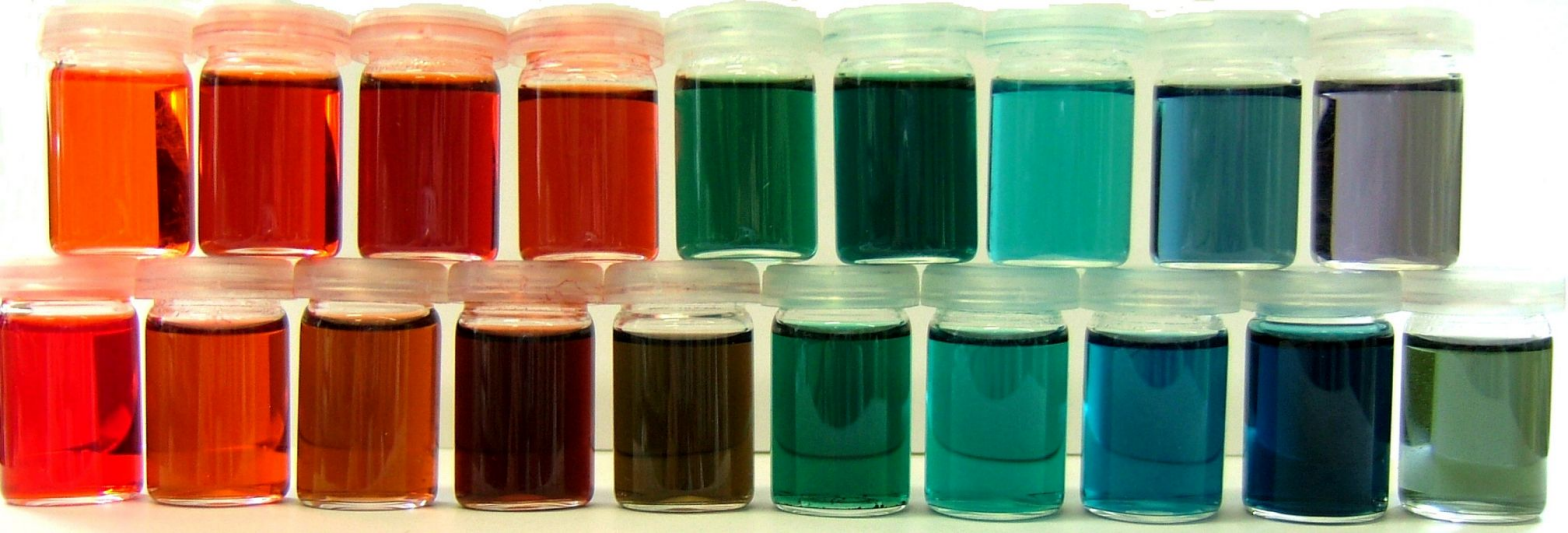
Broad spectrum of colors, created by mixing of green **2**, blue **6** and red **15**.

To avoid these effects, the concentrations of the monomer solutions had to be adapted. Each dye was diluted in acetone so as to show the same transparency and color brilliance. By mixing these solutions, a broad spectrum of colors can be created (Figure 5).

In this way, it is possible to create all color shades and nuances that are needed to replicate human irides, such as blue, green, brown and even gray. It is possible to print different color shades by using the synthesized functional dyes, which can then be polymerized afterwards [37–40]. Thus, a color design can be permanently included into polymeric materials, such as materials used for iris implants or other medical applications.

## Conclusion

In conclusion, a series of novel anthraquinone based polymerizable dyes has been synthesized by nucleophilic aromatic substitution reactions of anthraquinone derivatives with several amino alcohols. Depending on the moieties that were introduced, red-, blue- and green-colored dyes were prepared. After esterification with methacryloyl derivatives, polymerizable functionalities could be introduced. A broad color spectrum can be replicated by mixing these three dyes in different ratios and different concentrations. The dyes show no significant changes in their transmission spectra under sun-test conditions, and can be covalently emplaced into different copolymers. Thus, polymer materials that show no further diffusions of colored compounds out of the polymer networks were prepared. Containing two polymerizable functionalities, these monomeric dyes can also be used as cross-linking agents. Because of their stability, brilliance in color, and ability to cover a broad color spectrum, these monomers can be used for medical applications such as iris implants.

## Supporting Information

Supporting Information features detailed data on syntheses.

File 1 Experimental details.
