# Supplementary material for: Polymerization of novel methacrylated anthraquinone dyes
Source: Beilstein J Org Chem. 2013 Feb 28;9:453–9. doi: 10.3762/bjoc.9.48 (PMC3596084; doi:10.3762/bjoc.9.48)
Supplement: File 1 — Experimental details. [file Beilstein_J_Org_Chem-09-453-s001.pdf]

Supporting Information

for

Polymerization of novel methacrylated anthraquinone dyes

Christian Dollendorf, Susanne Katharina Kreth, Soo Whan Choi and Helmut Ritter*,§

Address: Institute for Organic and Macromolecular Chemistry II, Heinrich-Heine-Universität Düsseldorf, Universitätsstraße 1, 40225 Düsseldorf, Germany

§ Tel.: +49 (0)211-81 14760; fax: +49 (0)211-81 15840

* Corresponding author

Email: Helmut Ritter - H.Ritter@uni-duesseldorf.de

Experimental details

Measurements: ^{1}H NMR spectra were recorded on a Bruker Avance III – 300 and Bruker DRX500. UV-Vis data were recorded with a Nicolet UV 540-Spectrometer. FTIR spectra were recorded on a Fourier-transformation-IR spectrometer Type Nicolet FTIR-5SXB. GC-MS spectra are taken by Thermo Finnigan Trace DSQ (Dual-Stage Quadrupole) with electron spin ionization (ESI). EI spectra were recorded with a Varian MAT 311A spectrometer.

Materials: All solvents were purified and freshly distilled prior to use according to literature procedures.

Synthesis of 5,8-bis(4-(2-hydroxyethyl)phenylamino)-1,4-dihydroxyanthraquinone (1)

5.00 g (13.0 mmol) of 5,8-dichloro-1,4-dihydroxyanthraquinone was mixed with 5.57 g of (40.6 mmol) 2-(4-aminophenyl)ethanol and 4.24 g (29.9 mol) of disodium hydrogen phosphate under argon atmosphere in 13 mL of N-methyl-2-pyrrolidone (NMP). After stirring for 3 h at 150 °C, the mixture was heated to 180 °C for an additional 5 h. After cooling, 25 mL of methanol (MeOH) was added within 30 min at 65 °C. After heating under reflux for an additional hour, the mixture was cooled down to 55 °C for 1.5 h. After cooling, the mixture was filtered at room temperature and washed with 6 mL of MeOH/NMP (2:3), 50 mL of hot MeOH (65 °C) and 50 mL hot water (80 °C). The residue was dried at 70 °C, pestled, washed with ethyl acetate and dried in vacuo to give 3.07 g (6.01 mmol) of dark green 5,8-bis(4-(2-hydroxyethyl)phenylamino)-1,4-dihydroxyanthraquinone (**1**). Isolated yield = 46%. ^{1}H NMR (500 MHz, $CDCl_3$, rt) δ 7.13–7.38 (m, 12H), 5.23 (s, 2 H), 3.83 (t, 4 H), 2.83 (t, 4 H), 2.10 (s, 2 H) ppm; FTIR ν: 3314 (ν_{NH}), 3226 (ν_{OH}), 1607 ($\nu_{C=C}$), 1558 (ν_{NH}), 1489, 1450 ($\nu_{C=C}$), 1336 (δ_{OH}), 1041 (ν_{CO}), 973 916 (γ_{CH}), 819 (benzene-ring) cm^{-1}; EIMS *m/z*: 510 $[M]^+$, 479 $[M - 2(OH)]^+$, 378 $[M - OH - 2(=O) - 2((CH2)2OH)]^+$, 224 $[\text{anthraquinone} + OH]^+$; UV–vis (Chloroform) λ_{max}: 684, 636, 416 nm.

Synthesis of 5,8-bis(4-(2-methacryloxyethyl)phenylamino)-1,4-dihydroxyanthraquinone (2)

2 mL (13 mmol) of methacrylic anhydride was added to 1.00 g (2.00 mmol) of **1** and stirred at 110 °C for 40 min under microwave irradiation. 100 mL of water was added to the obtained residue and the crude product was purified by column chromatography (silica gel, ethyl acetate) to give 284 mg of green 5,8-bis(4-(2-methacryloxyethyl)phenylamino)-1,4-dihydroxyanthraquinone (**2**). Isolated yield = 22%. ^{1}H NMR (500 MHz, $CDCl_3$, rt) δ 7.15–7.26 (m, 12H, H), 6.03 (m, 2 H), 5.50 (m, 2 H), 4.48 (t, 4 H), 2.93 (t, 4 H), 1.98 (s, 6 H) ppm; FTIR ν: 3315 (ν_{NH}), 3220 (ν_{OH}), 1709 ($\nu_{C=C}$), 1559 (δ_{NH}), 1454 ($\nu_{C=C}$), 1342 (δ_{OH}), 1039 (ν_{CO}), 978 (γ_{CH}), 801 (benzene) cm^{-1}; EIMS *m/z*: 646 $[M]^+$, 560 $[M - \text{methylmethacrylate}]^+$, 547 $[M - \text{methylmethacrylate}]^+$, 99 $[\text{methylmethacrylate}]^+$. UV–vis (chloroform): λ_{max}: 684, 636, 416 nm.

Synthesis of 1,4-dichloroanthraquinone (3)

4.50 g (31 mmol) of 1,4-dichlorobenzene was added into a mixture of 3.50 g (17 mmol) phthalyl chloride, 0.5 g (3 mmol) phthalic anhydride, 0.4 g (7 mmol) sodium chloride and 3 g (22.7 mmol) aluminum chloride and heated for 6 h at 115 °C under reflux. After cooling, 30 mL of water was added carefully at 90 °C. The crude product was purified by steam distillation in order to remove remaining 1,4-dichlorobenzene, filtered and dried. 3.44 g of 2-(2,5-dichlorobenzoyl)benzoyl chloride was obtained, added to 30 g of concentrated sulfuric acid, and heated for 4 h at 150 °C. After cooling, 30 mL of water was added carefully at 80 °C. After cooling to room temperature, the precipitate was filtered, washed and dried at 120 °C to obtain 2.96 g (10.7 mmol) of greenish-brown 1,4-dichloroanthraquinone (**3**). Isolated yield = 60%. ^{1}H NMR (500 MHz, $CDCl_3$, rt) δ 8.13 (m, 2H), 7.73 (m, 2 H), 7.61 (s, 2H) ppm; FTIR ν: 3072 (ν_{CH}), 1671 ($\nu_{C=C}$), 1587 ($\delta_{C=O}$), 1548 1428 1370 ($\nu_{C=C}$), 1301 ($\nu_{C=O}$), 1246 1172 1129 985 (δ_{CH}), 717 (ν_{CCl}) cm^{-1}; GC–MS (acetone) *m/z*: 276 $[M]^+$, 248 $[M - 2{=}O]^+$, 241 $[M - Cl]^+$.

Synthesis of 1,4-bis(4-hydroxyphenylamino)anthraquinone (4)

1.27 g (4.57 mmol) of **3** was added to a mixture of 1.57 g (14.4 mmol) 4-aminophenol, 1.51 g (10 mmol) disodiumhydrogenphosphate and 5 mL NMP under argon atmosphere. The mixture was stirred for 3 h at 150 °C and for 12 h at 180 °C. 70 ml of MeOH was added within 30 min at 65°C, and the mixture was heated for an additional hour under reflux and 1.5 h at 55 °C. After cooling to room temperature, the crude product was filtered, washed with water and air dried to obtain 1.7 g (4 mmol) dark blue 1,4-bis(4-hydroxyphenylamino)anthraquinone (**4**). Isolated yield = 88%. ^{1}H NMR (500 MHz, $CDCl_3$, rt) δ 8.33 (m, 2 H), 7.70 (m, 2 H), 7.25 (m, 4 H), 7.15 (d, 2 H), 6.89 (m, 4 H) ppm; FTIR ν: 3218 (ν_{OH}), 1650 ($\nu_{C=C}$), 1593 1578 ($\nu_{C=O}$), 1555 ($\nu_{C=C}$), 1512 (ν_{NH}), 1445 1406 ($\nu_{C=C}$), 1362 (combination of δ_{OH} und ν_{CO}), 1245 (δ_{OH}), 1219 1165 (interaction of δ_{OH} und ν_{CO}), 1072 1026 (ν_{CO}) cm^{-1}; EIMS *m/z*: 422 [M]$^+$, 315 [M − aminophenyl]$^+$, 201 [aminophenyl]$^+$.

Synthesis of 1,4-bis(4-((2-hydroxyethyl)oxy)phenylamino)anthraquinone (5)

10 g (23.6 mmol) of **4** and 12.4 mL (176 mmol) of 2-bromoethanol were dissolved in 60 mL of 2N sodium hydroxide solution and heated for 4 h under reflux. 200 mL of water was added after cooling to room temperature. The precipitate was filtered and dried giving 7.9 g of **5**. Isolated yield = 66%. ^{1}H NMR (500 MHz, $CDCl_3$, rt) δ 8.32 (m, 2 H), 7.69 (m, 2 H), 7.24 (m, 2 H), 7.13 (m, 4 H), 6.88 (m, 4 H), 4.44 (t, 4 H), 4.17 (t, 4 H), 2.10 (s, 2 H) ppm; FTIR ν: 3326 (ν_{OH}), 2927 2970 (ν_{CH}), 1666 1555 ($\nu_{C=C}$), 1593 1576 ($\nu_{C=O}$), 1506 1456 1403 ($\nu_{C=C}$), 1363 (combination of δ_{OH} and ν_{CO}), 1243 (δ_{OH}), 1164 (interaction of δ_{OH} and ν_{CO}), 1071 1026 (ν_{CO}) cm^{-1}; EIMS *m/z*: 510 [M]$^+$, 465 [M − (CH2)2 − OH]$^+$, 45 [(CH2)2 − OH]$^+$; UV–vis (chloroform): λ_{max} = 408, 600, 644 nm.

Synthesis of 1,4-bis(4-((2-methacryloxyethyl)oxy)phenylamino)anthraquinone (6)

1 g (1.96 mmol) of **5**, 2 mL (38.3 mmol) of triethylamine and 1 mL (7.2 mmol) of methacryloyl chloride were mixed in 20 mL of acetonitrile for 30 min at room temperature. After the addition of 1 mL of ethylene glycol the mixture was stirred for another 30 min. 65 mL of water was added and the precipitate was filtered giving 1.2 g (1.86 mmol) of **6**. Isolated yield = 95%. ^{1}H NMR (500 MHz, $CDCl_3$, rt) δ 8.33 (m, 2 H), 7.70 (m, 2 H), 7.25 (m, 4 H), 7.15 (d, 2 H), 6.89 (m, 4 H), 6.10 (d, 2 H), 5.55 (d, 2 H), 4.45 (t, 4 H), 4.18 (t, 4 H), 1.91(s, 6 H) ppm; FTIR ν: 2926 2870 (ν_{CH}), 1709 ($\nu_{C=O}$), 1668 1608 ($\nu_{C=C}$), 1589 1572 1552 ($\nu_{C=O}$), 1511 1458 1403 ($\nu_{C=C}$), 1361 (combination of δ_{OH} and ν_{CO}), 1256 (δ_{OH}), 1164 (interaction of δ_{OH} und ν_{CO}), 1069 1015 (ν_{CO}) cm^{-1}; EIMS *m/z*: 646 [M]$^+$, 113 [ethylmethacrylate]$^+$, 69 [methacrylate – O]$^+$; UV–vis (chloroform) λ_{max}: 408, 600, 644 nm.

Synthesis of 1-chloro-4-((2-hydroxy-1,1-dimethylethyl)amino)anthraquinone (7) and 1,4-bis((2-hydroxy-1,1-dimethylethyl)amino)anthraquinone (8)

1 g (3.62 mmol) of **3** was added to a mixture of 1 g (11.3 mmol) 1,1-dimethyl-2-hydroxyethylamine and 1.18 g (8.31 mmol) of disodium hydrogen phosphate in 4 mL of NMP. The mixture was stirred for 3 h at 150 °C and for 12 h at 180 °C. 50 mL of MeOH was added within 30 min at 65 °C, with additional heating for another hour under reflux. After cooling at room temperature, 200 mL of water was added. The precipitate was filtered, washed with water and dried. The crude product was

purified by column chromatography (silica gel, hexane, ethyl acetate, 1:1) to obtain two products, red colored **7** and blue colored **8**. Isolated yields = 22% and 10%. ^{1}H NMR (**7**, 500 MHz, $CDCl_3$) δ 10.71 (s, 1H), 8.23 (dd, 1H, ^3J = 7.6 Hz, ^4J = 1.3 Hz), 8.14 (dd, 1H, ^3J = 7.6, Hz, ^4J = 1.3 Hz), 7.84 (m, 2H), 7.55 (d, 2H, ^3J = 1.9 Hz), 3.73 (m, 2H), 1.44 (s, 6H) ppm; EIMS *m/z* (**7**): 329 $[M]^+$, 312 $[M - OH]^+$, 289 $[M - (CH_2OH)]^+$, 256 $[M - C_4H_9O]^+$, 58 $[C_3H_6O]^+$, 44 $[C_2H_5O]^+$; UV–vis (**7**, chloroform) λ_{max}: 506 nm; ^{1}H NMR (**8**, 500 MHz, acetone-d_6) δ 11.39 (s, 2H), 8.20 (dd, 2H, ^3J = 5.8 Hz, ^4J = 3.3 Hz), 7.63 (m, 2H), 7.57 (s, 2H), 3.61 (s, 4H), 1.40 (s, 12H) ppm; EIMS *m/z* (**8**): 382 $[M]^+$, 351 $[M - (CH2OH)]^+$, 44 $[C2H5O]^+$.

Synthesis of 1-chloro-4-((2-methacryloxy-1,1-dimethylethyl)amino)anthraquinone (9)

0.644 g (1.96 mmol) of **7**, 2 mL (38.3 mmol) of triethylamine and 1 mL (7.2 mmol) of methacryloylchloride were mixed with 20 mL of acetonitrile and stirred for 30 min at room temperature. After the addition of 1 mL ethylene glycol the mixture was stirred for another 30 min. 300 mL of water was added and the precipitate was filtered to give 0.739 g (1.86 mmol) of red colored 1-chloro-4-((2-methacryloxy-1,1-dimethylethyl)amino)anthraquinone (**9**). Isolated yield = 95%. ^{1}H NMR (500 MHz, $CDCl_3$) δ 10.71 (s, 1H), 8.23 (dd, 1H, ^3J = 7.6 Hz, ^4J = 1.3 Hz), 8.14 (dd, 1H, ^3J = 7.6, Hz, ^4J = 1.3 Hz), 7.84 (m, 2H), 7.55 (d, 2H, ^3J = 1.9 Hz), 6.10 (d, 1H), 5.55 (d, 1H), 4.41 (s, 2H), 1.94 (s, 3H), 1.42 (s, 6H) ppm; EIMS *m/z*: 397 $[M]^+$, 328 $[M - \text{methacrylate}]^+$, 69 $[\text{methacrylate}]^+$; UV–vis (chloroform) λ_{max}: 505 nm.

Synthesis of 1,4-bis(4-((2-methacryloxy-1,1-dimethylethyl)amino)anthraquinone (10)

0.748 g (1.96 mmol) of **8**, 2 mL (38.3 mmol) of triethylamine and 1 mL (7.2 mmol) of methacryloylchloride were mixed with 20 mL of acetonitrile and stirred for 30 min at room temperature. After the addition of 1 mL of ethylene glycol the mixture was stirred for another 30 min. 300 mL of water was added and the precipitate was filtered to give 0.739 g (1.86 mmol) of blue colored **10**. ^{1}H NMR (500 MHz, acetone-d_6) δ 11.37 (s, 2H), 8.22 (dd, 2H, ^3J = 5.7 Hz, ^4J = 3.3 Hz), 7.56 (s, 2H), 7.62 (m, 2H), 6.11 (d, 2H), 5.61 (d, 2H), 4.41 (s, 4H), 1.92 (s. 6H), 1.40 (s, 12H) ppm; EIMS (acetone) *m/z*: 518 $[M]^+$, 449 $[M - \text{methacrylate}]^+$, 380 $[M - 2\text{methacrylate}]^+$, 69 $[\text{methacrylate} - O]^+$. UV–vis (chloroform) λ_{max}: 534, 574, 618 nm.

Synthesis of 1-((2-hydroxy-1,1-dimethylethyl)amino)anthraquinone (11)

3.15 g (13 mmol) of 1-chloroanthraquinone, 11.57 g (130 mmol) of 1,1-dimethyl-2-hydroxyethylamine and 1.83 g (12.88 mmol) of disodium hydrogen phosphate were mixed in 13 mL of DMSO under an argon atmosphere. The mixture was heated under reflux for 3 d at 100 °C and filtered after cooling to room temperature. The precipitate was washed with 300 mL of acetone, and the red solution was evaporated in vacuo. The viscous solution was added dropwise into 150 mL of cold water and the resulting precipitate was filtered. The crude product was purified by column chromatography (silica gel, dichloromethane, acetone, 4:1) to give 1.77 g of red colored **11**. Isolated yield = 50%. ^{1}H NMR (300 MHz, acetone-d_6) δ 10.35 (s, 1H), 8.24 (m, 2H), 7.85 (m, 2H), 7.54 (m, 3H), 3.74 (d, 2H, ^3J = 5.5 Hz), 1.51 (s, 6H) ppm; GC–MS (acetone) *m/z*: 296 $[M]^+$, 264 $[M - (CH2OH)]^+$, 222$[M - (C4H9O]^+$, 44 $[C2H5O]^+$; UV–vis (chloroform) λ_{max}: 500 nm.

Synthesis of 1-((2-methacryloxy-1,1-dimethylethyl)amino)anthraquinone (12)

1.77 g (6 mmol) of **11** was added to a solution of 5.6 mL (40 mmol) of triethylamine and 2.5 mL (24 mmol) of methacryloylchloride in 50 mL of acetonitrile. The mixture was stirred at room temperature for 30 min, and then 3 mL of ethylene glycol was added and stirred for additional 30 min. 100 mL of water was added and stirred for 8 h. The precipitate was filtered and purified by column chromatography (silica gel, hexane, acetone, 2:1) to give 2.16 g of red colored (5.8 mmol) **12**. Isolated yield = 98%. ^{1}H NMR (300 MHz, acetone-d_6) δ 10.36 (s, 1H), 8.22 (m, 2H), 7.84 (m, 2H), 7.58 (m, 3H), 6.1 (m, 1H), 5.64 (m, 1H), 4.41 (m, 2H), 1.91 (s, 3H), 1.61 (s, 6H) ppm; EIMS *m/z*: 363 $[M]^+$, 264 $[M - C5H7O2]^+$, 69 $[\text{methacrylate} - O]^+$; UV–vis (chloroform) λ_{max}: 505 nm.

Synthesis of 1-((1,3-dihydroxy-2-methylpropan-2-yl)amino)anthraquinone (13), 2-(hydroxymethyl)-2-methyl-2,3-dihydro-1*H*-anthra[2,1-*b*][1,4]oxazine-7,12-dione (14)

3.15 g (13 mmol) of 1-chloroanthraquinone was added to a mixture of 2-amino-2-methyl-1,3-propanediol and 1.83 g (12.88 mmol) of disodiumhydrogenphosphate in 13 mL of DMSO and heated under stirring to 100 °C for 3 d. The mixture was filtered, and the filter was washed with 100 mL of acetone. The solution was evaporated and added to 75 mL of cold water. After filtration and washing with water, the precipitate was dried and purified by column chromatography (silica gel, dichloromethane, acetone, 4:1) to give 0.46 g (1.5 mmol) of red colored **13** as the main product (isolated yield = 12%), and 33 mg of red colored **14** as a side product. ^{1}H NMR (**13**, 300 MHz, acetone-d_6) δ 10.44 (s, 1H), 8.29 (d, 1H, ^3J = 7.7 Hz), 8.18 (d, 1H, ^3J = 7.7 Hz), 7.85 (m, 2H), 7.57 (d, 2H, ^3J = 3.9 Hz), 7.51 (m, 1H), 3.88 (d, 4H, ^3J = 5.0 Hz), 1.46 (s, 3H) ppm; EIMS (**13**) *m/z*: 311 $[M^+]$, 280 $[M - CH3O]^+$. UV–vis (chloroform) λ_{max}: 502 nm.

^{1}H NMR (**14**, 500 MHz, acetone-d_6) δ 9.62 (s, 1H), 8.26 (d, 1H, ^3J = 7.7 Hz), 8.19 (d, 1H, ^3J = 7.7 Hz), 7.84 (m, 2H), 7.54 (d, 1H, ^3J = 8.1 Hz), 7.11 (d, 1H, ^3J = 8.1 Hz), 4.33 (d, 1H, ^3J = 10.7 Hz), 3.97 (d, 1H, ^3J = 10.7 Hz), 3.68 (m, 1H), 3.58 (m, 1H), 1.39 (s, 3H) ppm; EIMS (**14**) *m/z*: 309 $[M^+]$, 278 $[M - CH3O]^+$, 263 $[M - C2H6O]^+$; UV–vis (chloroform) λ_{max}: 502 nm.

Synthesis of 1-((1,3-dimethacryloxy-2-methylpropan-2-yl)amino)anthraquinone (15)

0.46 g (1.5 mmol) of **13** was added to a solution of 3 mL (20 mmol) of triethylamine and 0.7 mL (6 mmol) of methacryloylchloride in 50 mL of acetonitrile. The mixture was stirred at room temperature for 30 min, and then 3 mL of ethylene glycol was added and stirred for an additional 30 min. 100 mL of water was added and stirred for 8 h. The precipitate was filtered and purified by column chromatography (silica gel, hexane, ethyl acetate, 2:1) to give 0.52 g of red colored (1.2 mmol) 1-((1,3-dimethacryloxy-2,2-methylpropan-2-yl)amino)anthraquinone (**15**). Isolated yield = 75%. ^{1}H NMR (300 MHz, acetone-d_6) δ 10.49 (s, 1H), 8.24 (m, 2H), 7.87 (m, 2H), 7.63 (m, 3H), 6.17 (m, 2H), 5.66 (m, 2H), 4.53 (m, 4h), 1.93 (s, 6H), 1.71 (s, 3H) ppm; EIMS *m/z*: 447 $[M]^+$, 348 $[M - C5H7O2]^+$, 262 $[M - C9H12O4]^+$, 69 $[\text{methacrylate} - O]^+$; UV–vis (chloroform) λ_{max}: 492 nm.

Copolymerization of THFMA, HEMA, EGDMA and anthraquinone dyes 9 and 10

2.13 g (16 mmol) of 2-hydroxyethylmethacrylate (2-HEMA), 867 mg (5 mmmol) of tetrahydrofurfuryl methacrylate (THFMA) and 10.0 mg (0.05 mmol) of ethylene glycol dimethacrylate (EGDMA) were mixed under an argon atmosphere. After that, 2.10 mg (0.008 mmol) of V 65 and 5 mg or 6.5 mg (0.013 mmol) of the anthraquinone dyes **9** or **10** were added and stirred under an argon atmosphere. The solution was filled into the form and heated at 100 °C for 24 h. FTIR (Diamond) ν*:* 3413 (ν_{OH}), 2880 (ν_{CH}), 1720 ($\nu_{C=O}$), 1450 (δCH), 1387 (δOH), 1271 (ν_{CO}), 1223 (ν_{CO}), 1019 (ν_{CO}), 1153 (ν_{CO}) cm^{-1}.
